# Imaging dataset of fresh hydrous plants obtained by field-emission scanning electron microscopy conducted using a protective NanoSuit

**DOI:** 10.1371/journal.pone.0232992

**Published:** 2020-05-11

**Authors:** Sayuri Takehara, Yasuharu Takaku, Masatsugu Shimomura, Takahiko Hariyama

**Affiliations:** 1 Preeminent Medical Photonics Education & Research Center, Institute for NanoSuit Research, Hamamatsu University School of Medicine, Higashi-ku, Hamamatsu, Shizuoka, Japan; 2 Departments of Bio- and Material Photonics, Chitose Institute of Science and Technology, Chitose, Hokkaido, Japan; Pennsylvania State Hershey College of Medicine, UNITED STATES

## Abstract

Although scanning electron microscopy (SEM) can generate high-resolution images of nanosized objects, it requires a high vacuum to do so, which precludes direct observations of living organisms and often produces unwanted structural changes. It has previously been reported that a simple surface modification gives rise to a nanoscale layer, termed the “NanoSuit”, which can keep small animals alive under the high vacuum required for field-emission scanning electron microscopy (FE-SEM). We have previously applied this technique to plants, and successfully observed healthy petals in a fully hydrated state using SEM. The flower petals protected with the NanoSuit appeared intact, although we still lack a fundamental understanding of the images of other plants observed using FE-SEM. This report presents and evaluates a rich set of images, acquired using the NanoSuit, for a taxonomically diverse set of plant species. This dataset of images allows the surface features of various plants to be analyzed and thus provides a further complementary morphological profile. Image data can be accessed and viewed through *Figshare* (https://doi.org/10.6084/m9.figshare.c.4446026.v1).

## Introduction

Scanning electron microscopy (SEM) enables us to observe the nanoscale fine structure of biological materials with high resolution. To obtain these high-resolution images, the SEM specimen chamber requires a high vacuum to prevent electron scattering and use the electron beam effectively. This creates one of the harshest known environments for biological specimens, as approximately 70–80% of living tissue is water. Therefore, biological specimens routinely require pre-treatments such as chemical fixation prior to dehydration, freeze-drying, critical point drying, or metal coating to avoid sample damage and to stabilize the structures for SEM [1]. Unfortunately, these procedures preclude the direct observation of living organisms and often produce unwanted structural changes, even in fixed specimens. To overcome this limitation of conventional SEM, technologies such as low-vacuum scanning electron microscopy [2] and environmental scanning electron microscopes (ESEM) [3] have been developed, which require reduced vacuum but result in inferior resolution with a typical maximum magnification of 2,000x.

We have previously reported that a simple surface modification on living tissues can produce a thin external layer, which we call a “NanoSuit.” This allows small animals to survive in the high vacuum required for field-emission scanning electron microscopy (FE-SEM) [4–6]. The NanoSuit uses the natural extracellular substance (ECS) that covers some organisms, or in some cases an added substance mimicking the ECS and polymerizes it using electron beam or plasma irradiation [7]. Because the NanoSuit maintains the integrity of the organism’s surface under the conditions required for high-resolution SEM imaging, it enables the use of sophisticated observation methods to study biological materials at magnifications exceeding 10,000x [8]. We have now applied this technique to flower petals and examined their response to high-vacuum environments [9]. The results show that, with a NanoSuit but without any other pre-treatment, the overall morphology of cherry petals is well-preserved after SEM imaging, suggesting that the natural substances on the petal surface behave like animal ECSs and form a NanoSuit upon electron beam irradiation [9]. Despite this progress, we still lack comparable results for other fresh hydrous plants with the NanoSuit method and FE-SEM. Most previously reported FE-SEM data is for samples prepared using conventional methods. The primary objective of this report is to develop image profiles of plants using the NanoSuit for specimens from different plant taxonomic groups, thus enabling further comparative investigations. Furthermore, some morphological variations are also presented in addition to the well-preserved images, to demonstrate potential applications of the presented data; e.g. examining the protective properties of the plant surface to extreme environments.

## Materials and methods

### Experimental organisms

Specimens of *Hyophila propagulifera* Broth. (Bryophyta, Pottiaceae), *Hydrangea macrophylla* (Thunb.) Ser. (Hydrangeaceae), and *Taraxacum officinale* sect. Taraxacum (Asteraceae) were collected from the campus of Hamamatsu University School of Medicine (137° 72’ E, 34° 70’ N). The Green laver, *Monostroma nitidum* Wittrock (Chlorophyta, Monostromataceae) was collected from Lake Hamana (137° 55’ E, 34° 70’ N).

Line strains of other living plants, *Asplenium trichomanes* L. (Polypodiopsida, Aspleniaceae), *Dendrobium Snow baby* × *Snow angel* cv. Angel Baby (Orchidaceae), *Pinus thunbergii* Parl. (Pinaceae), *Lavandula angustifolia* Mill. (Lamiaceae), *Rosa × hybrida* (Rosaceae), and *Tagetes erecta* L. (Asteraceae) were collected and cultured at room temperature, with sufficient watering prior to experimental use.

The petals of three varieties of Japanese flowering cherry, *Prunus yedoensis* Matsum. cv. Somei-Yoshino (Rosaceae), *Prunus spachiana* (Lavall‚e ex H.Otto) Kitam. f. spachiana (Rosaceae), and *Phlox subulata* L. (Polemoniaceae) were collected from Gotenba city (138° 52’ E, 35° 17’ N).

### Microscopy

FE-SEM was conducted using a JEM-7100F (JEOL) instrument operated at acceleration voltages of 1.0 kV. The vacuum level of the observation chamber was 10^−3^–10^−6^ Pa. The value was about 10^−6^ Pa around the gun of the electron beam and about 10^−4^–10^−5^ Pa around the observation stub. The detector for secondary electrons was a signal from a lower detector. In addition, the working distances were 6–10 mm, the aperture size φ was 100 μm, the scan speed for each beam was 10–15 frames/second, and the dwell time for taking one image was ca. 37 seconds. The beam current was about 100 μA and the beam irradiation density and dose were approximately 2.65 x 10^17^ /m^2^ to 9.56 x 10^18^ /m^2^, depending on the observation conditions.

### Preparation for standard SEM

**Fig 1:**
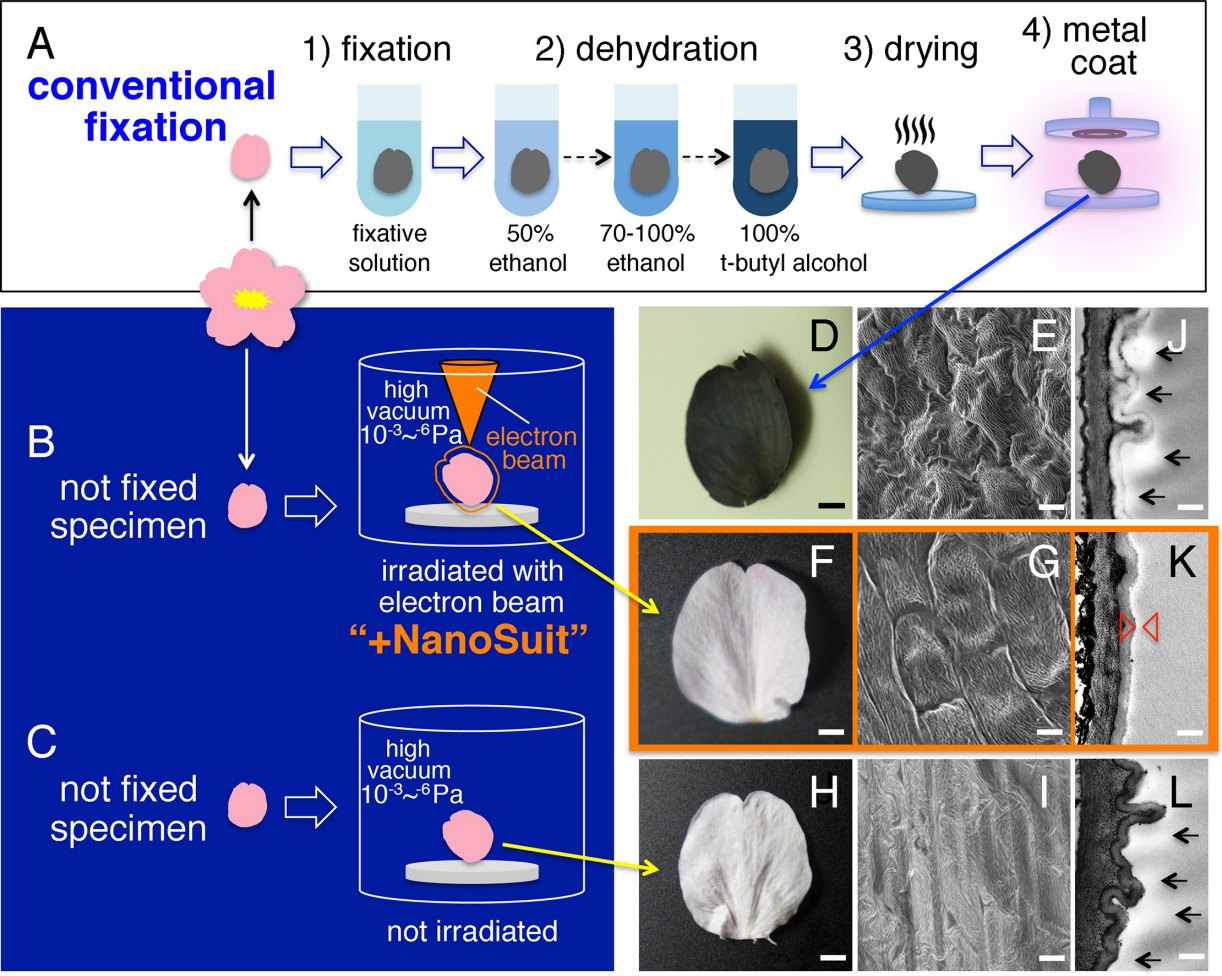
Preparation of specimens for SEM and subsequent optical, SEM, and TEM images. Schematic of specimens (A) prepared with conventional fixation methods, (B) treated by the NanoSuit method, and (C) placed in the SEM observation chamber, but without concurrent electron beam radiation. Images of cherry blossom petals obtained by (D, F, H) light microscopy and (E, G, I) SEM. SEM images were taken by 1,000x (E, G, I) with acceleration voltages of 5.0 kV (E) or 1.0 kV (G, I) at working distances of 7.1 mm (E), 6.6 mm (G), or 7.2 mm (I) under vacuum conditions of 1.6^−3^ Pa (E), 1.9^−4^ Pa (G), or 1.4^−3^ Pa (I). (J, K, L) Transmission electron microscopy (TEM) images of petal cross sections. The arrows in J and L indicate the position of the surface material covering the petals (white layers). The white layer between the arrowheads in K indicates the newly formed NanoSuit. Scale bars are (D, F, H) 2 mm, (E, G, I) 10 μm, and (J, K, L) 300 nm.

For conventional SEM observations, plant specimens were prefixed with 2% glutaraldehyde in 0.1 M of phosphate buffer (pH 7.4) and postfixed in 1% OsO_4_ in the same buffer. The specimens were then dehydrated through a graded series of ethanol, transferred to t-butyl alcohol, freeze dried (JFD300, JEOL), and coated with an ultra-thin layer of OsO_4_ (PMC-5000, Meiwa) (Fig 1A).

### Sample preparation for FE-SEM by the NanoSuit method

**Fig 2:**
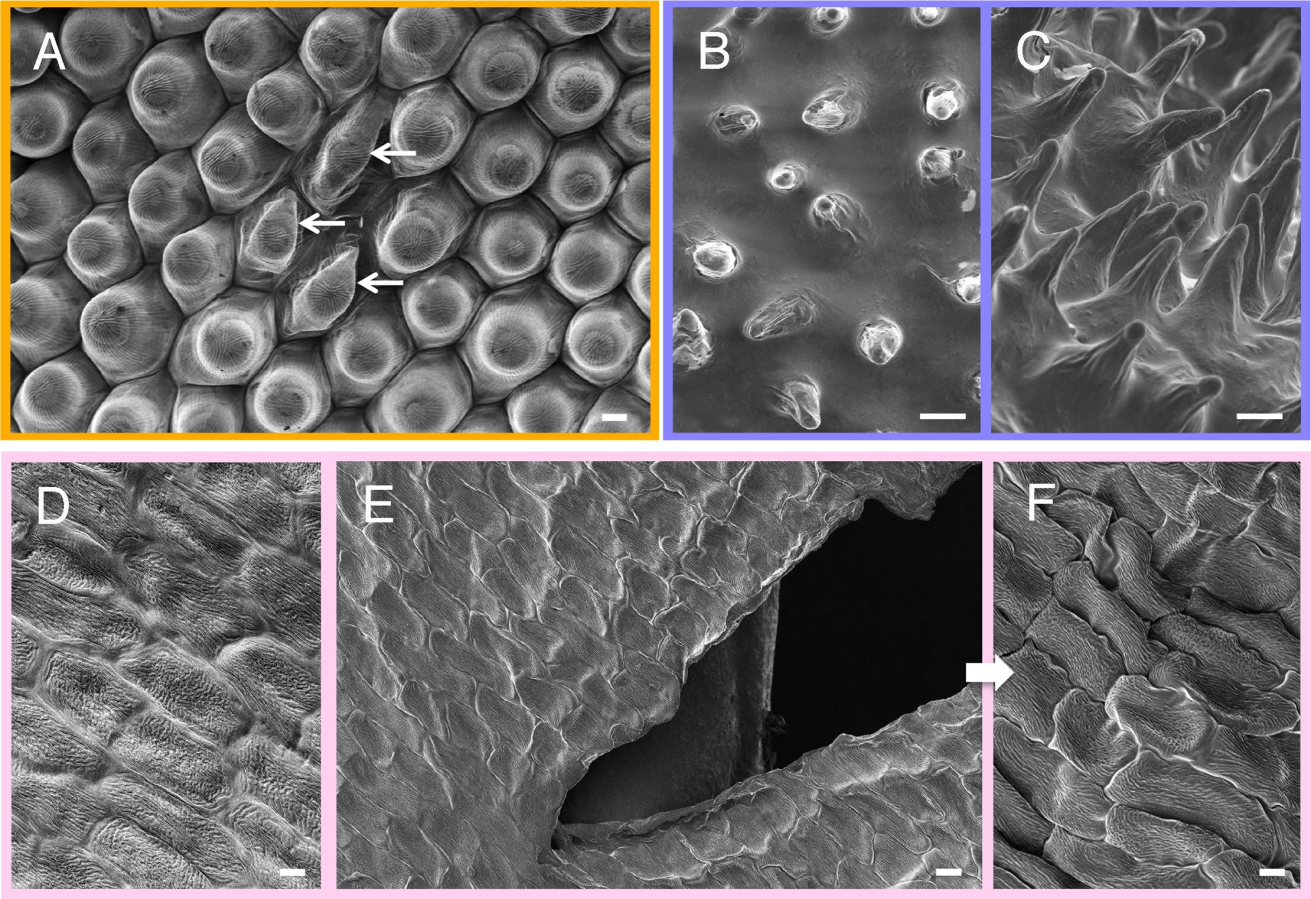
SEM images showing morphological variations in specimens prepared using the NanoSuit method. (A) Image demonstrating that some cells (indicated by arrows) appear to have collapsed on the surface of the petal. On the same petal, some areas (B) secrete a large volume of the natural surface substances, whereas other areas (C) have a small amount of the substances on the surface. (D) Image of the healthy petal surface. (E) Image after specimen was cut with a scalpel to create a wound on the surface. (F) Image of the changed structure after wounding. SEM images were taken by 300x (E), 500x (A), or 1,000x (B–D, F) with acceleration voltage of 1.0 kV at working distances of 7.3 mm (A), 6.9 mm (B), 7.8 mm (C), 7.1 mm (D), 10.1 mm (E), or 10.2 mm (F) under vacuum conditions of 1.6^−3^ Pa (A), 1.5^−3^ Pa (B), 1.4^−3^ Pa (C), 1.6^−3^ Pa (D), 1.3^−3^ Pa (E), or 5.8^−4^ Pa (F). Scale bars are (A, B, C, D, F) 10 μm, (E) 20 μm.

The workflow of the NanoSuit method for hydrous plants is summarized in Fig 1B and described here. Only healthy plant specimens were selected for experiments. Specifically, specimens having bruises on the surface were rejected because damaged specimens were more likely to lose weight under high vacuum [9], which would result in deformation of the fine structures during observation (cf. Fig 2D–2F). Electron beam irradiation with low magnification (20–30x) was then conducted to the entire surface of the specimens, and the areas where a NanoSuit formed were used for SEM without any conventional pre-treatments, such as chemical fixation, dehydration, or ultrathin coating of electrically conductive materials [4, 9].

### Preparation for transmission electron microscopy

Prior to any other treatment, some petal specimens were placed in the SEM observation chamber for 20 minutes either with (cf. Fig 1K), or without (cf. Fig 1L) electron beam irradiation. All specimens were then treated by the following procedure. For transmission electron microscopy (TEM), specimens were prefixed in 2% glutaraldehyde in 0.1 M cacodylate buffer (pH 7.4), and then postfixed in 1% OsO_4_ in the same buffer. The dehydrated specimens were embedded in an Epon-Araldite mixture. Ultra-thin sections (approximately 70 nm) were cut (ULTRACAT OmU_4_, REICHERT-JUNG) vertical to the surface and stained with 2% uranyl acetate followed by 0.4% lead citrate (cf. Fig 1J).

### Data records

The image dataset presented here contains the raw images of 218 EM files in an 8-bit TIFF format (271 dpi) at magnifications ranging from 100x to 10,000x. It includes the plants treated with the NanoSuit method and the specimens prepared using conventional fixation methods, as summarized in Table 1. The datasets for various plant specimens described in this paper are available at the following *Figshare* locations:

- *Monostroma nitidum* Wittrock (Chlorophyta, Monostromataceae)
  (https://doi.org/10.6084/m9.figshare.7471337.v3),
- *Hyophila propagulifera* Broth. (Bryophyta, Pottiaceae)
  (https://doi.org/10.6084/m9.figshare.7477718.v2),
- *Asplenium trichomanes* L. (Polypodiopsida, Aspleniaceae)
  (https://doi.org/10.6084/m9.figshare.7477766.v2),
- *Dendrobium Snow baby* × *Snow angel* cv. Angel Baby (Orchidaceae)
  (https://doi.org/10.6084/m9.figshare.7477778.v2),
- *Pinus thunbergii* Parl. (Pinaceae)
  (https://doi.org/10.6084/m9.figshare.7477787.v2),
- *Prunus yedoensis* Matsum. cv. Somei-Yoshino (Rosaceae)
  (https://doi.org/10.6084/m9.figshare.7477829.v2),
- *Prunus spachiana* (Lavall‚e ex H.Otto) Kitam. f. spachiana (Rosaceae)
  (https://doi.org/10.6084/m9.figshare.7477847.v2),
- *Phlox subulata* L. (Polemoniaceae)
  (https://doi.org/10.6084/m9.figshare.7477874.v2),
- *Hydrangea macrophylla* (Thunb.) Ser. (Hydrangeaceae)
  (https://doi.org/10.6084/m9.figshare.7477877.v2),
- *Lavandula angustifolia* Mill. (Lamiaceae)
  (https://doi.org/10.6084/m9.figshare.7477886.v2),
- *Rosa × hybrida* (Rosaceae)
  (https://doi.org/10.6084/m9.figshare.7477901.v2),
- *Tagetes erecta* L. (Asteraceae)
  (https://doi.org/10.6084/m9.figshare.7477907.v2), and
- *Taraxacum officinale* sect. Taraxacum (Asteraceae)
  (https://doi.org/10.6084/m9.figshare.7477910.v2).

**Table 1:** Overview of the raw image files.

	petal	Petal (variation)	leaf	Leaf (variation)	thallus	total
***Monostroma nitidum***					18	18
***Hyophila propagulifera***			8	2		10
***Asplenium trichomanes***			14			14
***Dendrobium Snow baby* × *Snow angel* cv. Angel**	10					10
***Pinus thunbergii***			18			18
***Prunus yedoensis* Matsum. cv. Somei-Yoshino**	8	21				29
***Prunus spachiana* f. spachiana**	4	5				9
***Phlox subulata***	7	5				12
***Hydrangea macrophylla***	8	5				13
***Lavandula angustifolia***	13	5	13	1		32
***Rosa × hybrida***	4	4	9			17
***Tagetes erecta* L. (Asteraceae)**	13	3				16
***Taraxacum officinale* sect. Taraxacum**	7	13				20
**total**	74	61	62	3	18	218

21 files of the “petal (variation)” for *Prunus yedoensis* Matsum. cv. Somei-Yoshino include 11 files of images prepared using conventional fixation methods.

## Results and discussion

Fig 1 provides a schematic overview of the two sample preparations used: (1) one example of conventional fixation methods for SEM observation (Fig 1A) and (2) NanoSuit protection for direct SEM imaging (Fig 1B). A control sample without any conventional fixation or NanoSuit protection is also included as Fig 1C. Contrary to the cherry blossom petals prepared by conventional methods (Fig 1D and 1E), those protected with a NanoSuit appeared intact (Fig 1F and 1G). This agrees with our previous reports on the differences between images collected using conventional fixation and NanoSuit methods [4, 8, 9]. In addition to preserving the specimens, the NanoSuit method is also much faster than conventional methods. Because the conventional fixation methods include various treatments, they typically take two days (Fig 1A), while the NanoSuit method only takes a few minutes to complete (Fig 1B).

Fig 1D and 1E show typical images of cherry blossom petals prepared using conventional fixation methods. These methods include dehydration and/or drying, which is the most common procedure used to prepare samples to ensure stability under a high vacuum. However, under these conditions, specimens inevitably shrink (cf. f_petal_19–29 (conventional), *In* https://doi.org/10.6084/m9.figshare.7477829.v2) since approximately 70–80% of living tissue is water. Moreover, conventional methods require an ultrathin coating of an electrical conductor such as gold, palladium, platinum, or osmium [1]. If the specimens are not coated with electrically conductive materials, they exhibit electrostatic charging, which prevents satisfactory imaging during SEM observation [6]. Conversely, specimens protected with the NanoSuit showed no electrostatic charging, even without any electrically conductive coating (Fig 1F and 1G; cf. f_petal_1–8, *In* https://doi.org/10.6084/m9.figshare.7477829.v2). We have previously reported that substances on the petal surface behaves like wax and its ability to serve as a protective barrier is improved upon electron beam irradiation, which turns the substance into the NanoSuit [9]. However, when the specimens were placed in the SEM observation chamber for 20 minutes at an identical vacuum level, but without concurrent electron beam radiation (Fig 1C and 1H), subsequent SEM observations revealed that the surface structures shrank and exhibited electrostatic charging (Fig 1I). Cross-sectional transmission electron microscopy (TEM) images (Fig 1J–1L) showed that all three specimens had an extra layer of material covering their surface (white layers indicated by arrows in Fig 1J and 1L). However, for specimens irradiated the surface layer was thinner (Fig 1K), which suggests that the surface material was polymerized by the electron beam and a NanoSuit was thus successfully formed. Together, these results indicate that, because of the protective thin film formed by the NanoSuit, this method enables better observation of structural details to be observed and studied.

We compared the images of all specimens prepared using the NanoSuit method, which includes not only fragile petals but also some examples of stable leaf surface structures (e.g. https://doi.org/10.6084/m9.figshare.7477886.v2). The electron micrographs sometimes show morphological variations, even on the same specimen (Fig 2A; cf. l_petal_14–16, *In* https://doi.org/10.6084/m9.figshare.7477907.v2). Some possible causes for these variations are discussed herein. For example, Fig 2B shows an area that is rich in the natural substances that are secreted on the petal surface (cf. j_petal_11–13 (rich surface material), *In* https://doi.org/10.6084/m9.figshare.7477886.v2), while Fig 2C shows an area where relatively less of these substances were secreted (cf. j_petal_1–4, *In* https://doi.org/10.6084/m9.figshare.7477886.v2). These images suggest that, where a large volume of the natural substances is secreted, a rather “thick” NanoSuit is formed, which prevents the underlying fine structure from being properly imaged [6]. By contrast, where the natural substances are too thin to adequately protect specimens from dehydration, partially disrupted structures are observed in the SEM images (cf. j_petal_14–18 (morphological variation), *In* https://doi.org/10.6084/m9.figshare.7477886.v2).

Fig 2D–2F shows a typical example of the structural changes which occurred with the NanoSuit method, depending on the mechanical stress experienced. When we made a cut with a knife (Fig 2E), it induced deformation of the surrounding fine structures (Fig 2D versus 2F; cf. f_petal_1–8 versus f_petal_9–18 (morphological variation), *In* https://doi.org/10.6084/m9.figshare.7477829.v2) [9]. Although the electron beam irradiations used in the present investigations did not induce conformational changes, these specimens experienced with the mechanical stress sometimes shrunk and/or collapsed within a few seconds during the dwell time for taking images. Thus the NanoSuit formed from the natural surface substances did not serve as an adequate barrier against such a large wound, and water was lost from the crack in the NanoSuit. This suggests that further improvements to the NanoSuit are needed for it to more effectively protect living specimens against extreme environments.

## Conclusion

The protective barrier for living tissues that is provided by the NanoSuit technique is a desirable tool for enabling observation of living organisms. The polymerized thin film NanoSuit applied here plays an important role in keeping the samples hydrous in the FE-SEM, thus permitting high magnification and high vacuum to be used to observe the fine structures in biological specimens with high resolutions. The dataset presented here provides FE-SEM images for various fresh hydrous plants, allowing users to inspect the three-dimensional configuration of fine surface structures in plants under living conditions. The method represents a simpler, less time-consuming procedure compared to conventional specimen treatment methods, and should be suitable for numerous applications, not only in biology but also in many other fields of science.
